# High-Density Lipoprotein Therapy in Stroke: Evaluation of Endothelial SR-BI-Dependent Neuroprotective Effects

**DOI:** 10.3390/ijms22010106

**Published:** 2020-12-24

**Authors:** Alexy Tran-Dinh, Angélique Levoye, David Couret, Lauriane Galle-Treger, Martine Moreau, Sandrine Delbosc, Camille Hoteit, Philippe Montravers, Pierre Amarenco, Thierry Huby, Olivier Meilhac

**Affiliations:** 1 Institut National de la Santé et de la Recherche Médicale (INSERM), UMR 1148, Bichat-Claude Bernard Hospital, Paris University, 75018 Paris, France; alexy.trandinh@aphp.fr (A.T.-D.); sandrine.delbosc@inserm.fr (S.D.); hoteit.camille@gmail.com (C.H.); pierre.amarenco@aphp.fr (P.A.); 2 Department of Anesthesiology and Critical Care, Bichat-Claude Bernard Hospital, AP-HP. Nord, Paris University, 75018 Paris, France; philippe.montravers@aphp.fr; 3 Paris Centre de Recherche Cardiovasculaire (PARCC), Institut National de la Santé et de la Recherche Médicale (INSERM), Paris University, 75015 Paris, France; angleique.levoye@inserm.fr; 4 Institut National de la Santé et de la Recherche Médicale (INSERM), UMR 1188, Diabète Athérothrombose Réunion Océan Indien (DéTROI), Reunion Island University, 97411 Saint-Denis de La Réunion, France; david.couret@chu-reunion.fr; 5 Centre Hospitalo-Universitaire de La Réunion, 97411 Saint-Denis de La Réunion, France; 6 Institute of Cardiometabolism and Nutrition (ICAN), Institut National de la Santé et de la Recherche Médicale, UMR 1166, Sorbonne University, 75006 Paris, France; lauriane.galle@gmail.com (L.G.-T.); martine.moreau@upmc.fr (M.M.); thierry.huby@upmc.fr (T.H.); 7 Institut National de la Santé et de la Recherche Médicale (INSERM), UMR 1152, Bichat-Claude Bernard Hospital, Paris University, 75018 Paris, France; 8 Department of Neurology, Bichat-Claude Bernard Hospital, AP-HP. Nord, Paris University, 75018 Paris, France

**Keywords:** high-density lipoprotein, SR-BI, stroke, blood-brain barrier

## Abstract

High-density lipoproteins (HDLs) display endothelial protective effects. We tested the role of SR-BI, an HDL receptor expressed by endothelial cells, in the neuroprotective effects of HDLs using an experimental model of acute ischemic stroke. After transient intraluminal middle cerebral artery occlusion (tMCAO), control and endothelial SR-BI deficient mice were intravenously injected by HDLs or saline. Infarct volume and blood-brain barrier (BBB) breakdown were assessed 24 h post tMCAO. The potential of HDLs and the role of SR-BI to maintain the BBB integrity was assessed by using a human cellular model of BBB (hCMEC/D3 cell line) subjected to oxygen-glucose deprivation (OGD). HDL therapy limited the infarct volume and the BBB leakage in control mice relative to saline injection. Interestingly, these neuroprotective effects were thwarted by the deletion of SR-BI in endothelial cells and preserved in mice deficient for SR-BI in myeloid cells. In vitro studies revealed that HDLs can preserve the integrity of the BBB in OGD conditions, and that this effect was reduced by the SR-BI inhibitor, BLT-1. The protection of BBB integrity plays a pivotal role in HDL therapy of acute ischemic stroke. Our results show that this effect is partially mediated by the HDL receptor, SR-BI expressed by endothelial cells.

## 1. Introduction

Acute ischemic stroke (AIS) is a major cause of mortality and disability. Intravenous thrombolysis by recombinant tissue plasminognen activator (rt-PA) is the mainstay of AIS management within 4.5 h from the onset of neurologic symptoms [1]. Endovascular mechanical thrombectomy and intra-arterial thrombolysis represent promising approaches that are still under evaluation in clinical trials [2]. However, less than 5% of patients suffering from AIS are treated by intravenous thrombolysis. Novel neuro-vasculoprotective agents are necessary to prevent deleterious secondary effects of ischemia and to improve the benefits of recanalization therapies. Among risk factors of stroke, high-density lipoprotein—cholesterol (HDL-C) levels were shown to be inversely associated with the risk of recurrent ischemic stroke [3]. We have shown that HDLs are dysfunctional in acute stroke condition and display altered protective effects on cerebral endothelial cells [4]. These results support a supplementation strategy using functional therapeutic HDLs. We have previously demonstrated that HDLs isolated from human plasma and injected intravenously reduced the mortality and the infarct volume in a rat model of embolic stroke [5]. We also observed that intravenous infusion of HDLs limited hemorrhagic transformation induced by recombinant tissue plasminogen activators (rtPA) in two models of rat cerebral ischemia [6]. In both studies, the blood-brain barrier (BBB) was suggested to play a pivotal role in the protective effects of HDLs. The BBB is a continuous layer composed of endothelial cells surrounded by pericytes and astrocyte endfeets that represents an active interface between the blood stream and the central nervous system. Cerebral ischemia leads to BBB disruption and to a loss of brain homeostasis [7]. Protection of the BBB integrity represents a major target in the treatment of AIS. 

HDLs are a family of complex particles composed of multiple proteins including apolipoprotein A-I (apoA-I, the most abundant protein), phospholipids and cholesterol esters. They are characterized by their ability to transport cholesterol in excess from peripheral tissues back to the liver. In addition, HDLs display multiple beneficial effects on the endothelium such as anti-oxidant, anti-apoptotic, anti-inflammatory, anti-thrombotic and anti-protease properties [8].

Scavenger receptor class B type 1 (SR-BI) is a major HDL receptor highly expressed in the liver, where it mediates the selective uptake of HDL cholesteryl esters. SR-BI is also expressed by endothelial cells, including those composing the BBB [9] and is involved in the endothelial protective effects of HDLs [10]. The interaction between HDL particles and endothelial SR-BI induces nitric oxyde synthase activation [11] leading to inhibition of apoptosis [12] and mobilization of bone marrow-derived endothelial progenitor cell [13]. In addition, SR-BI signaling pathway induces endothelial migration and proliferation [14], inhibits platelets aggregation [15] and is involved in HDL transcytosis across the endothelium [16].

In this study, we demonstrate the neuroprotective effects of HDLs in a mouse model of AIS. Moreover, we show the involvement of endothelial SR-BI in the protection of the BBB by HDLs both in vivo using an endothelial SR-BI KO model (SR-BI *^flox/flox^*; Tie2-Cre) and in vitro, in a cellular model of BBB (human brain microvascular endothelial cell line subjected to normoxia and ischemia/reperfusion conditions).

## 2. Results

### 2.1. HDLs Limit the Infarct Volume in a Mouse Model of Acute Cerebral Ischemia/Reperfusion

**Figure 1:**
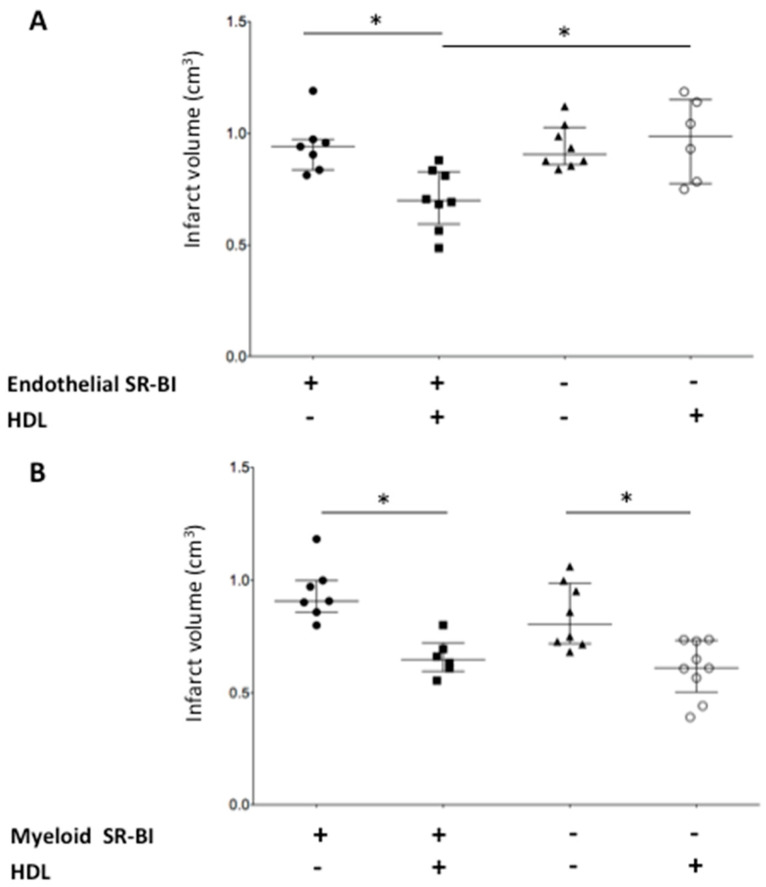
Role of endothelial SR-BI in the neuroprotective effects of HDLs in AIS. Infarct volume was assessed on coronal brain slices by TTC staining after a 4 h intraluminal occlusion of the middle cerebral artery followed by 20 h of reperfusion. (**A**) In SR-BI *^flox/flox^* mice, intracarotid infusion of HDLs limited the infarct volume by 25% relative to saline-injected mice (* *p* < 0.05). The neuroprotective effects of HDLs were thwarted by the deletion of endothelial SR-BI in SR-BI *^flox/flox^*/Tie2-Cre (* *p* < 0.05). (**B**) The neuroprotective effects of HDLs were not mediated by the expression of SR-BI in myeloid cells. In SR-BI *^flox/flox^* mice, intracarotid infusion of HDLs limited the infarct volume by 28% compared to saline infusion (* *p* < 0.05). The neuroprotective effects of HDLs were maintained despite the deletion of myeloid SR-BI in SR-BI *^flox/flox^*/LysM-Cre mice (* *p* < 0.05).

Cerebral ischemia/reperfusion was performed by a transient intraluminal middle cerebral artery occlusion (MCAO). In control mice (SR-BI *^flox/flox^*), intracarotid injection of HDLs at the reperfusion, after a 4 h MCAO, limited the infarct volume by 25% relative to mice injected with saline (0.70 (0.60–0.83) vs. 0.94 (0.84–0.97) cm^3^, respectively, *p* < 0.05) (Figure 1A).

### 2.2. Endothelial SR-BI Is Involved in the Neuroprotective Effects of HDLs

To test the role of SR-BI in HDL-mediated neuroprotection, we subjected endothelial SR-BI KO mice (SR-BI *^flox/flox^*/Tie2-Cre) to a transient MCAO followed by reperfusion. The deletion of SR-BI in endothelial cells thwarted the neuroprotective effects of HDLs. Indeed, we observed in the HDL-treated group that the infact volume was higher in SR-BI *^flox/flox^*/Tie2-Cre compared to SR-BI *^flox/flox^* mice (0.99 (0.78–1.15) vs. 0.70 (0.60–0.83) cm^3^, respectively, *p* < 0.05) (Figure 1A). Interestingly, in the group of mice infused with saline, endothelial SR-BI deletion did not impact on the infarct volume (0.94 (0.84–0.97) vs. 0.91 (0.86–1.03) cm^3^, in SR-BI *^flox/flox^* and SR-BI *^flox/flox^*/Tie2-Cre mice respectively, NS) (Figure 1A). 

### 2.3. Myeloid SR-BI Does Not Mediate HDL Neuroprotective Effects

Tie-2-mediated expression of the Cre recombinase leads to deletion of floxed SR-BI allele in endothelial cells but also to some extent in myeloid cells [17]. The expression of SR-BI is well documented in monocytes/macrophages [18] and has also been reported in neutrophils [19]. These myeloid immune cells play a critical role in acute cerebral ischemia [20]. To investigate the possibility that the results observed in SR-BI *^flox/flox^*/Tie2-Cre on the protective effects of HDLs in our model of ischemic stroke might be related, at least in part, to SR-BI deletion in myeloid cells, we generated SR-BI *^flox/flox^*/LysM-Cre mice to specifically inactivate SR-BI in the myeloid lineage. By contrast with the results observed in SR-BI *^flox/flox^*/Tie2-Cre, the protective effects of HDLs on the infarct volume were preserved in SR-BI *^flox/flox^*/LysM-Cre mice and did not affect the protective effects of HDLs on the infarct volume (0.65 (0.60–0.72) vs. 0.61 (0.50–0.73) cm^3^, in SR-BI *^flox/flox^* and SR-BI *^flox/flox^*/LysM-Cre mice respectively, NS) (Figure 1B). This result strongly supports that the neuroprotective effects of HDLs are mainly mediated by endothelial SR-BI. 

### 2.4. HDLs Limit the BBB Leakage via Endothelial SR-BI

**Figure 2:**
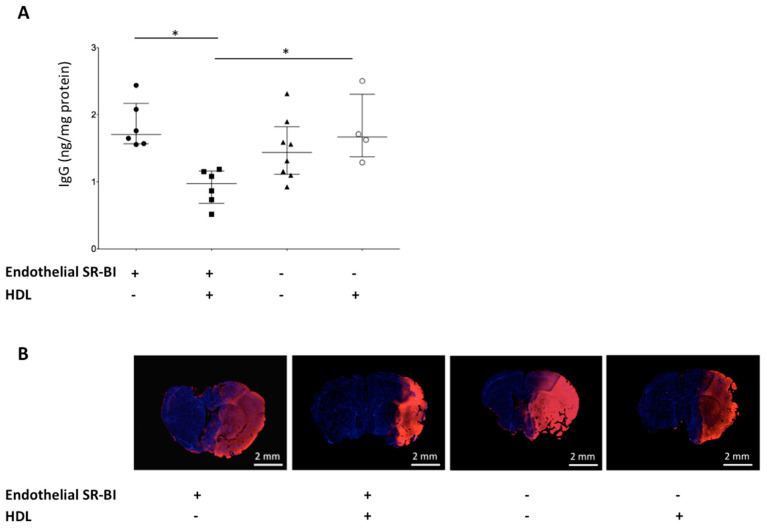
Role of endothelial SR-BI in the BBB integrity after AIS. (**A**) In SR-B *^flox/flox^* mice, intracarotid infusion of HDLs decreased extravasation of blood-borne immunoglobulins G across the BBB compared to saline infusion (* *p* < 0.05). The protective effects of HDLs on the BBB integrity were thwarted by the deletion of endothelial SR-BI in SR-BI *^flox/flox^*/Tie2-Cre mice (* *p* < 0.05). (**B**) BBB leakage was qualitatively assessed by blood-borne albumin extravasation into the brain parenchyma. Immunofluorescence staining of albumin (in red) on coronal brain slices 24 h after middle cerebral artery occlusion (MCAO) followed by reperfusion showed a smaller albumin positive area in SR-BI *^flox/flox^* (“endothelial SR-BI +”) mice treated with HDLs vs. saline. The deletion of endothelial SR-BI in SR-BI *^flox/flox^*/Tie2-Cre mice (“endothelial SR-BI −”) did not prevent albumin extravasation after HDL infusion.

BBB breakdown was assessed by the extravasation of blood-borne IgGs into the brain parenchyma. Infusion of HDLs in SR-BI *^flox/flox^* mice limited the BBB leakage by decreasing the amount of IgGs detected within the brain parenchyma compared to mice infused with saline (1.0 (0.7–1.2) vs. 1.7 (1.6–2.2) ng/mg of total protein, respectively, *p* < 0.05). The deletion of endothelial SR-BI impeded HDL protection regarding the extravasation of circulating IgGs into the brain parenchyma (1.7 (1.4–2.3) vs. 1.0 (0.7–1.2) ng/mg of tissue protein, in SR-BI *^flox/flox^*/Tie2-Cre and SR-BI *^flox/flox^* mice, respectively, *p* < 0.05) (Figure 2A). Of note, among mice infused with saline after tMCAO, endothelial SR-BI deletion did not impact on the extravasation of IgGs across the BBB (1.7 (1.6–2.2) vs. 1.4 (1.1–1.8) ng/mg of tissue protein, in SR-BI *^flox/flox^*/Tie2-Cre and SR-BI *^flox/flox^* mice respectively, NS). Qualitative assessment of BBB leakage by immunofluorescent staining of albumin on coronal brain sections showed a smaller area of blood-borne albumin extravasation through the BBB in SR-BI *^flox/flox^* mice treated with HDLs compared to saline injection. Deletion of SR-BI in endothelial cells blunted the protective effects of HDLs (Figure 2B).

### 2.5. HDLs Display Protective Effects on the Integrity of a Cellular Model of BBB Under Ischemia/Reperfusion Conditions

**Figure 3:**
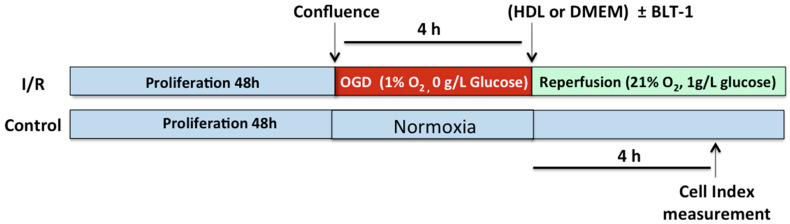
General scheme of experiments using a human cellular model of BBB in normoxia and ischemia/reperfusion conditions. hCMEC/D3 cells were grown in a 8-well L-plate (iCELLigence system) for 48 h to reach confluence. Cells were then subjected to normoxic or ischemia/reperfusion (I/R) conditions. I/R was mimicked by a 4 h OGD followed by addition of 21% oxygen and 1 g/L glucose. At reperfusion, hCMEC/D3 were incubated with HDLs or culture medium alone (DMEM) with or without increasing concentrations of SR-BI inhibitor, BLT-1. Cell impedance was monitored in real time using the RTCA Software during the whole experiment.

To confirm the in vivo protective effects of HDLs on the BBB integrity, we used an in vitro model consisting of human brain microvascular endothelial cell line (hCMEC/D3) cultured onto gold microelectrodes, allowing real time monitoring of cell-cell interactions, reflected by changes in impedance that is low at baseline. hCMEC/D3 used as a human model of BBB for pathogenic studies [21]. The integrity of this cellular model of BBB was assessed by measuring impedance variations. The general scheme of the experiment is presented in Figure 3.

**Figure 4:**
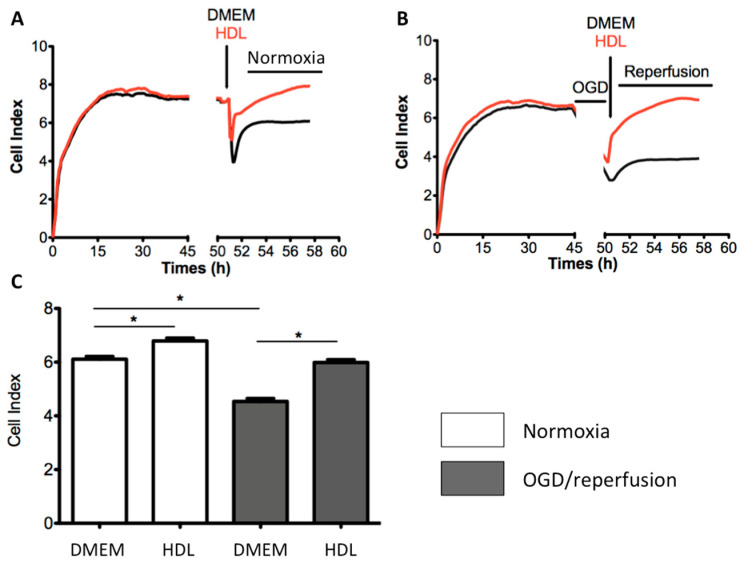
Role of HDLs on the integrity of a human cellular model of BBB in normoxic and ischemia/reperfusion con-ditions. Cell impedance (converted into cell index) was monitored in real time, and plots were produced using the RTCA Software. (**A**,**B**). At confluence, hCMEC/D3 were subjected to normoxia or to a 4 h oxygen-glucose deprivation (OGD) followed by reperfusion. (**C**) Cell index values were compared 4 h after incubation with or without HDLs in normoxia and OGD/reperfusion conditions. HDLs restored endothelial integrity in normoxic and OGD/reperfusion conditions compared to DMEM alone (* *p* < 0.0001).

At confluence, hCMEC/D3 were subjected to normoxia (Figure 4A) or to a 4-h oxygen-glucose deprivation (OGD) followed by reperfusion (Figure 4B) with or without HDL incubation. In normoxic conditions, incubation of hCMEC/D3 with HDLs for 4 h increased the cell index relative to saline (6.80 ± 0.08 vs. 5.91 ± 0.1, respectively, *p* < 0.0001) (Figure 4C). In ischemia/reperfusion conditions, OGD led to a decrease of cell index relative to baseline (4.54 ± 0.11 vs. 6.11 ± 0.11, respectively, *p* < 0.0001) (Figure 4C). HDL addition to hCMEC/D3 at reperfusion maintained the BBB integrity as compared to incubation with culture medium alone (5.99 ± 0.10 vs. 4.54 ± 0.11, respectively, *p <* 0.0001) (Figure 4C).

### 2.6. Endothelial SR-BI Is Involved in the Protective Effects of HDLs on the Integrity of a Cellular Model of BBB in Ischemia/Reperfusion Conditions

**Figure 5:**
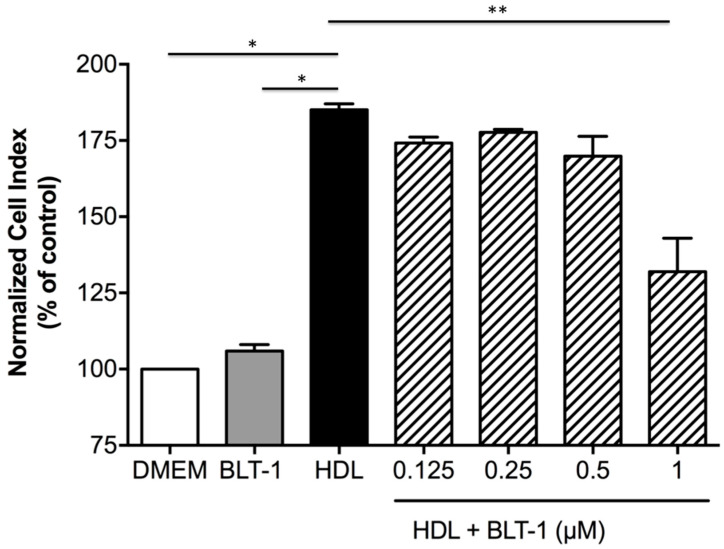
Role of endothelial SR-BI in the protective effects of HDLs on the integrity of a human cellular model of BBB in ischemia/reperfusion conditions. The role of endothelial SR-BI on the protective effects of HDLs on the integrity of hCMEC/D3 cell line BBB model in OGD/reperfusion conditions was assessed by the comparison of the normalized cell index (expressed as % of control) 4 h after incubation of hCMEC with or without increasing concentrations of SR-BI inhibitor BLT-1 from 0.125 to 1 µM. HDLs display protective effects on the integrity of the BBB compared to DMEM alone (* *p* < 0.05). Addition of 1 µM BLT-1 inhibited the protective effects of HDLs on BBB integrity (** *p* < 0.05), whereas 1 µM BLT-1 alone did not decrease cell index.

To study the role of SR-BI in the protective effects of HDLs on the BBB integrity, we used an antagonist of SR-BI, BLT-1 [22]. In hCMEC/D3 subjected to ischemia/reperfusion conditions and incubated with HDLs, the addition of 1µM BLT-1 partially counteracted the protective effects of HDLs on BBB integrity (132 ± 10.9 vs. 185.1 ±1.9, respectively, *p* < 0.05). No change in the cell index was observed in the presence of 1µM BLT-1 alone indicating no toxic effect of BLT-1 on cell viability (Figure 5). This suggests that endothelial protective effects of HDLs in ischemia/reperfusion conditions are mediated by SR-BI. 

## 3. Discussion

In this study, we show that the endothelial HDL receptor, SR-BI is involved in the neuroprotective effects of HDL therapy in a mouse model of acute cerebral ischemia/reperfusion. In vitro, HDLs maintain the endothelial integrity in a cellular model of human BBB subjected to oxygen-glucose deprivation (OGD). We demonstrated that endothelial SR-BI plays a pivotal role in HDL protective effects. 

This is the first study showing the neuroprotective effects of HDL therapy on cerebral ischemia/reperfusion in mice. HDL infusion after a 4 h intraluminal tMCAO reduced the infarct volume by 25% relative to saline injection. This result confirmed the neuroprotective effects of HDLs observed by Lapergue et al. in a rat model of stroke [5,6]. Our results also suggest that HDLs strengthen the BBB integrity in AIS in vivo, and in OGD conditions, in vitro. Our in vivo results showed that HDL therapy decreased the extravasation of blood-borne IgGs across the BBB into the brain parenchyma after stroke. In vitro, the incubation of HDLs with a cellular model of human blood-brain barrier maintained cell-cell interactions after OGD followed by reperfusion. Our group also showed that HDL therapy reduced hemorrhagic complications associated with tissue plasminogen activator treatment in a rat model of ischemic stroke, supporting the vasculoprotective action of HDLs on the BBB under ischemic conditions [6]. There are different HDL receptors such as ABCA1, ABCG1 and SR-BI. Several studies have suggested that hCMEC/D3 cell line express SR-BI [23,24], and our immunofluorescence analysis confirmed this result (data not shown). It is not excluded that other receptors such as ATP Binding Cassette (ABC) transporters, reported to be expressed by hCMEC/D3, may be involved in the protective effects of HDLs on the BBB [25].

In our mouse model of acute cerebral ischemia/reperfusion, endothelial SR-BI deletion suppressed the protective effects of HDL therapy. In endothelial SR-BI deficient mice, myeloid immune cells also exhibit decreased levels of SR-BI [17,26], which could have participated in the blunted effect of HDLs. We thus performed the same experiment with mice exclusively deleted for myeloid SR-BI. We did not observe any effect of myeloid SR-BI deletion on the neuroprotective action of HDLs, which supports the primary role of endothelial SR-BI. Interestingly, the protecting role of HDLs in immune cell-induced vascular inflammation has been usually attributed to the ABCA1 receptor [27,28,29,30].

Interestingly, the deletion of endothelial SR-BI in mice infused with saline did not modify the infarct volume. This result suggests that the observed neuroprotective effects were not mediated by endogenous mouse HDLs, but required the interaction between functional exogenous HDLs and endothelial SR-BI. Indeed, AIS could lead to dysfunctional endogenous HDLs which display blunted protective effects [4]. Besler et al. showed that HDLs from patients suffering from acute coronary syndrome have reduced endothelial anti-inflammatory potential. Their capacity to stimulate endothelial repair decreased in a model of carotid electric injury, due to an impaired HDL-dependent endothelial nitric oxyde production [31]. In our model, we thus infused a high dose of exogenous HDLs (80 mg/kg) isolated from healthy volunteers that doubled HDL plasma concentration. This dose was used from Shaw et al. [32].

Our study has several limitations. First, we used HDLs isolated from human plasma but not from mouse plasma due to their limited total blood volume. Second, these HDLs are heterogeneous and their composition may vary from batch to batch. In future studies, the evaluation of reconstituted HDLs with a determined composition in proteins and lipids would be necessary to provide mechanistic insights and to translate into clinical applications. Third, we have not evaluated the molecular mechanisms at the cellular level involved in the neuroprotective effects of HDL interaction with endothelial SR-BI. HDLs display pleiotropic effects including antioxidant, anti-apoptotic, anti-inflammatory, anti-thrombotic or anti-proteolytic properties that account for their protective action on endothelial cells [8]. HDLs have been shown to regulate the vascular tone via their interaction with SR-BI, leading to the stimulation of endothelial release of nitric oxide via an activation of the endothelial nitric oxide synthase [33,34,35]. HDLs also have protective anti-inflammatory properties for the endothelium by inhibiting the expression of adhesion molecules, ICAM-1 and VCAM-1 [36], and by modulating the NF-κB pro-inflammatory intracellular pathway [36]. From a perspective view, it would be interesting to assess the role of SR-BI in HDL uptake by cerebral endothelial cells. Indeed, Rohrer et al. showed HDL transcytosis by aortic endothelial cells in a SR-BI dependent pathway. Inhibition of SR-BI with small interfering RNA limited HDL binding by 50% and transcytosis by 35–40% in aortic endothelial cells [16]. Moreover, we have previously shown in a rat model of stroke that HDLs penetrated the infarct area and were taken up by endothelial cells and astrocytes [5]. Fourth, we did not evaluate the functionality and the lipid composition of endothelial cells from endothelial SR-BI KO mice. We cannot exclude that endothelial SR-BI KO mice could display cell membrane abnormalities, which could be responsible for the lack of protective effect of HDLs. Indeed, Munoz-Vega et al. showed that HDLs could improve endothelial function by supplying lipids to these cells, via an SR-BI dependent internalization of HDL particles [37]. Moreover, SR-BI has been shown to act as a plasma membrane cholesterol sensor, which induces intracellular signaling necessary for HDL activation of endothelial nitric oxyde synthase, for the migration of endothelial cells in culture, and for HDL-induced angiogenesis in vivo [38]. As a result, endothelial SR-BI KO mice may exhibit altered membrane lipid balance resulting in major endothelial cell dysfunction, which could not be rescued by HDLs after ischemic insult. However, this did not impact on the infarct size without HDLs in endothelial SR-BI KO mice, which was similar to that of wild-type mice. 

In conclusion, our study shows the pivotal role of endothelial SR-BI in the neuroprotective effects of HDL therapy in stroke by preserving the BBB integrity.

## 4. Methods

### 4.1. Mouse Models of Endothelial and Myeloid Deletion of SR-BI

SR-BI *^flox/flox^* mice [39] were crossed with Tie2-Cre mice which express the Cre recombinase under the direction of the receptor tyrosine kinase Tek (Tie2) promoter/enhancer [40] to delete SR-BI in endothelial cells (SR-BI *^flox/flox^*/Tie2-Cre). Alternatively, SR-BI *^flox/flox^* mice were crossed with LysM-cre mice expressing Cre under the endogenous lysozyme M promoter (*Lyz2 ^tm1(cre)Ifo^*; Jackson Laboratory) to generate animals deficient for SR-BI in the myeloid cell lineage (SR-BI *^flox/flox^*/LysM-Cre) [17]. 

### 4.2. Preparation of HDLs

Blood samples were obtained from healthy volunteers with informed consent, according to French Law L.1243-3 modified by articles R1243-49 to 56, requiring the declaration of “Biobanking and preparation of cells and tissues from human body for research purpose” to MESR (French higher education and research ministry), Inserm (French National Institute for Health and Medical Research), and ANSM (French National Agency for Medicines and Health Products Safety) with the following references. Inserm: C 19-23, IDRCB: 2019-A01137-50, and MESR: DC-2016-2614. 

Lipoproteins were isolated from a pool of EDTA-treated plasma by ultracentrifugation. Briefly, plasma density was adjusted to *d* = 1.22 with KBr and overlaid with KBr saline solution (*d* = 1.063). Ultracentrifugation was performed at 100,000× *g* for 20 h at 10 °C. The density of the bottom fraction containing HDLs was adjusted to 1.25 with KBr and overlaid with KBr saline solution (*d* = 1.22). The HDL fraction (top layer) was recovered as a single band and was rinsed with saline and concentrated using a centrifugal concentrating device (cutoff 10 kDa; Vivascience, Stonehouse, UK). All fractions were desalted either by dialysis against saline or by centrifugation and three washes with saline. Protein concentration was determined using the Bicinchoninic Acid (BCA) method (BCA Protein Assay Kit, Thermo Scientific Pierce, Waltham, MA, USA).

### 4.3. Mouse Model of Focal Cerebral Ischemia/Reperfusion

**Figure 6:**
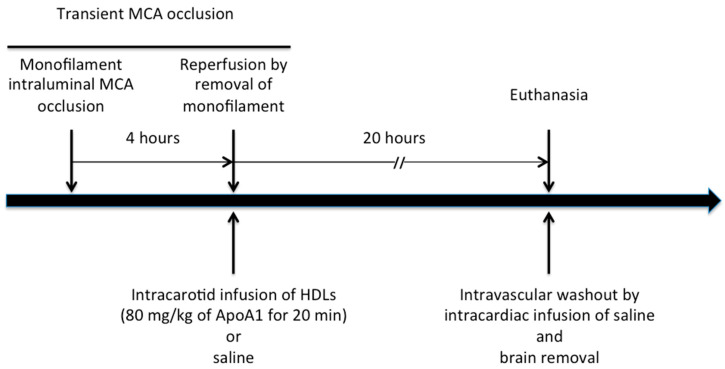
In vivo experimental protocol. Transient middle cerebral artery (MCA) occlusion was performed by intraluminal occlusion of MCA for 4 h followed by reperfusion. At the start of reperfusion, an intracortid infusion of HDLs at a dose of 80 mg/kg of ApoA1 for 20 min or saline for control mice was performed. Mice were euthanized after 20 h of reperfusion with a concomitant intravascular washout by intracardiac infusion of saline followed by brain removal.

Animal experiment protocols were approved by the Institutional Animal Care and Use Committee of the INSERM-University Paris Diderot (2012-15/698-0098). The investigation conformed to the European directive 2010/63/EU revising directive 86/609/EEC on the protection of animals used for scientific purposes. Mice (male; 10 week-old) were anesthetized with isoflurane under spontaneous ventilation and analgesia was performed with subcutaneous injection of buprenorphine (0.05 mg/kg). Body temperature was maintained at 37 °C with a heating pad. Cerebral ischemia/reperfusion was induced by a 4-h transient intraluminal middle cerebral artery occlusion (MCAO) by introducing a 7-0 silicon-coated monofilament (Doccol Corporation, Sharon, MA, USA) via the right common carotid to dramatically decrease cerebral blood flow at the bifurcation of the right MCA and the right internal carotid. After removing the monofilament, reperfusion was allowed for the next 20 h. Mice were excluded if there was a failure during the in vivo procedure (Figure 6).

### 4.4. In Vivo Experimental Protocol

Intracarotid infusion of HDLs (80 mg/kg of apoA-I for 20 min) or saline was randomly performed at the onset of cerebral reperfusion after a 4 h MCAO. Briefly, a catheter (SAI Infusion technologies mouse tail vein catheter) was introduced into the common carotid artery after removing the monofilament in order to infuse approximatively 250 mL of an 8 mg/mL HDL solution. Control mice were injected with the same volume of saline. The catheter was then removed and the common carotid ligated. Randomization was performed by a computer generated-list. Syringes containing HDL or saline were prepared upstream and then made indistinguishable. Analyzes were performed by other researchers than the experimenter.

### 4.5. Infarct Volume BBB Leakage

Twenty-four hours after the stroke onset, mice were euthanized. Intravascular washout was performed by intracardiac infusion of saline. The brain was removed and cut into 1 mm-coronal sections using a brain matrix mold. Infarct volume was measured using Image J software from coronal brain sections stained with 2,3,5-triphenyltetrazolium chloride (TTC). Results were expressed in cm^3^.

BBB leakage was evaluated by two methods: first by quantifying of the extravasation blood-borne immunoglobulins G (IgG) through the BBB into the brain parenchyma in ischemic brain tissue homogenates (Mouse ELISA Kit-Abcam, Cambridge, UK). Results were expressed in ng/mg of tissue protein; and second by a qualitative evaluation by immunostaining for mouse albumin in brain sections. Brain samples were fixed with 3.7% paraformaldehyde and embedded in paraffin. Immunofluorescence analysis was performed on 5-μm-thick sections using an anti-albumin antibody (Ab 34807, Abcam). Briefly, the sections were blocked with 10% goat serum and incubated overnight at 4 °C with primary antibodies (1:200 dilution). We included nonimmune rabbit IgG in each set of experiments as the primary antibody to test the specificity of the signal and used Alexa-Fluor 594 as secondary antibody. Immunostaining was analyzed with a Nanozoomer digital slide scanner (Hamamatsu).

### 4.6. In Vitro Assessment of the BBB Integrity

The human brain endothelial cell line (hCMEC/D3) was kindly provided by Dr Couraud (Institut Cochin, Paris, France). The expression of SR-BI by hCMEC/D3 was assessed using a polyclonal SR-BI antibody (Novus Biologicals, Centennial, CO, USA). hCMEC/D3 were grown in a 8-well L-plate of an iCELLigence system (Ozyme, Saint-Cyr-l’École, France). At confluence (48 h after seeding), cells were subjected to normoxic (21% O_2_) or ischemia/reperfusion conditions. Ischemia was mimicked by a 4 h oxygen–glucose deprivation (OGD). OGD was obtained by using Dulbecco’s Modified Eagle’s Medium (DMEM) without glucose (Gibco) versus DMEM 1 g/L glucose for non-OGD conditions (normoxic condition). hCMEC/D3 were exposed to oxygen deprivation (1% O_2_) by using hypoxia chamber (Galaxy 48R, Eppendorf, Hamburg, Germany) with oxygen sensor attached for monitoring of oxygen concentration in the chamber. After 4 h of OGD, cells were returned to normal conditions (21% O_2_ and 1 g/L of glucose). At the reperfusion, hCMEC/D3 were treated with or without 400 µg/mL HDL solution in DMEM. Impedance values were monitored in real time and converted to cell index (CI) values using the RTCA Software. This technology measures the flow of electrons transmitted between gold microelectrodes in the presence of an electrically conductive solution such as tissue culture medium. Adherent cells interfere with the interaction between the electrodes and thus impede the flow of electrons. This impedance is expressed in arbitrary units called the CI, which depends on the strength of adhesion of the cells to the substrate covering the plate [41]. CI values were compared 4 h after reperfusion and incubation of hCMEC/D3 with or without HDLs in normoxic and OGD conditions. 

The role of endothelial SR-BI in the protective effects of HDLs was assessed with or without increasing concentrations of SR-BI inhibitor BLT-1 (Sigma-Aldrich, Saint Louis, MI, USA) from 0.125 to 1 µM. 

### 4.7. Sample Size Calculation

The study was designed with 80% power to detect a relative 25% difference in cerebral infarct volume between groups. Statistical testing was performed at the 2-tailed level of 0.05 using a t test. Based on preliminary data indicating that mean infarct volume at 24 h after stroke was 0.95 cm^3^ (SD +/− 0.13), sample size calculation indicated five mice per group.

### 4.8. Statistical Analysis

For in vivo experiments, data are presented as median with interquartile range. Data were analyzed by a Kruskal-Wallis test followed by a Dunn’s multiple comparison test if *p* < 0.05. A 2-tailed value of *p* < 0.05 was considered significant. For in vitro experiments, results are expressed as mean ± SEM and were analyzed by repeated ANOVA followed by a Turkey’s multiple comparison test if *p* < 0.05. A 2-tailed value of *p* < 0.05 was considered significant. Statistical analysis were performed using Prism v5.0 (Graphpad Software). 

## Data Availability

The data presented in this study are available on request from the corresponding author.

## Abbreviations

AIS	Acute ischemic stroke
ApoA1	apolipoprotein A-I
BBB	Blood-brain barrier
HDL	High-density lipoprotein
IgG	Immunoglobulin G
MCAO	Middle cerebral artery occlusion
OGD	oxygen-glucose deprivation
rt-PA	recombinant tissue plaminogen activator
SR-BI	Scavenger receptor class B type I
TTC	2,3,5-triphenyltetrazolium chloride
